# Non-Canonical Binding of Nelfinavir in HIV-1 Protease Variants Reveals Structural Mechanisms of Antiretroviral Resistance

**DOI:** 10.3390/v18070701

**Published:** 2026-06-25

**Authors:** Christian Cadena-Cruz, Marcio De Avila-Arias, Fabio Guzmán, Mariana Pérez, María Angelica Zuluaga, Elkin Navarro Quiroz, Alejandro Angulo, Luz Elena Prieto Garcerant, Hector Rodríguez Rojas, Dinno Alberto Fernández Chica, Guillermo Cervantes, Jose Luis Villarreal-Camacho

**Affiliations:** 1 Programa de Bacteriología, Universidad Libre Seccional Barranquilla, Barranquilla 081007, Colombia; 2 Grupo GIBAC (Grupo de Investigación Básicas y Clínicas), Fundación Universitaria San Martín (FUSM), Puerto Colombia 081007, Colombia; 3 Grupo de Investigación en Biomedicina, Facultad de Ciencias Básicas Biomédicas, Universidad Metropolitana, Barranquilla 081007, Colombia; 4 Programa de Microbiología, Universidad Libre Seccional Barranquilla, Barranquilla 081007, Colombia; fabioa-guzmanv@unilibre.edu.co; 5 Independent Researcher, Barranquilla 080020, Colombia; marianadelatorre@uninorte.edu.co (M.P.); mariazuluagac@gmail.com (M.A.Z.); alejoangulo22@hotmail.com (A.A.); guicerva@uninorte.edu.co (G.C.); 6 Facultad de Ciencias Básicas y Biomédicas, Universidad Simón Bolívar, Barranquilla 081007, Colombia; elkin.navarro@unisimon.edu.co; 7 Especialización en Medicina Interna, Universidad Libre Seccional Barranquilla, Barranquilla 081007, Colombia; luze-prietog@unilibre.edu.co (L.E.P.G.); hectora-rodriguezr@unilibre.edu.co (H.R.R.); dinnoa.fernandezc@unilibre.edu.co (D.A.F.C.); 8 Programa de Medicina, Universidad Libre Seccional Barranquilla, Barranquilla 081007, Colombia; josel.villarrealc@unilibre.edu.co

**Keywords:** antiretroviral, resistance, mutations, non-canonical binding, interactions

## Abstract

Background: Antiretroviral resistance-associated mutations, within the broader context of HIV-1 genetic variability, represent a growing challenge for HIV-1 control, highlighting the need for continuous molecular surveillance and mechanistic understanding of drug resistance. This study aimed to characterize mutations in the pol gene associated with resistance to protease inhibitors and to explore their structural implications. Methods: Viral RNA was extracted from plasma samples of HIV-positive patients, and a 266 bp fragment of the HIV-1 pol gene was amplified by RT-PCR and sequenced using the Sanger method. Sequences showing ≥98% homology were aligned and analyzed using MEGA v11 and the Stanford HIV Drug Resistance Database to identify resistance-associated mutations, while viral subtypes were determined using COMET, jpHMM-HIV, and STAR tools. Amino acid sequences were used for structural modeling with AlphaFold, followed by molecular docking with Nelfinavir using the CB-Dock2 server. Results: Four samples exhibited resistance-associated profiles, including high-level, intermediate, and low-level resistance, with one isolate showing high-level resistance to multiple protease inhibitors. Structural analyses revealed that Nelfinavir preferentially binds to alternative hydrophobic cavities rather than the canonical catalytic site, lacking direct interactions with the Asp25/Asp25′ dyad. Conclusions: These findings suggest a structural mechanism of resistance based on non-canonical ligand binding that may impair effective protease inhibition.

## 1. Introduction

Acquired immunodeficiency syndrome (AIDS), caused by the human immunodeficiency virus (HIV), was first recognized in 1981 [1]. Early clinical manifestations of the infection were characterized by opportunistic infections and the appearance of purplish lesions associated with Kaposi’s sarcoma, which prompted significant scientific interest in understanding the disease and developing effective strategies to control its progression.

The first antiretroviral agents capable of suppressing viral replication were introduced in 1987, initially as monotherapy regimens, most notably zidovudine (AZT) [2]. However, the rapid evolution and adaptability of HIV soon led to the emergence of resistance-associated mutations, as evidenced by viral isolates from patients undergoing long-term AZT treatment as early as 1989 [3].

The approval of the first protease inhibitors in 1996 marked a major breakthrough, enabling the development of combination antiretroviral therapy (cART) [4]. This therapeutic strategy transformed HIV infection from a fatal disease into a manageable chronic condition, leading to significant improvements in immune function, reductions in viral RNA levels, regression of opportunistic infections such as Kaposi’s sarcoma, increased CD4+ T cell counts, and decreased mortality rates [5,6,7,8,9]. Consequently, antiretroviral therapy became the cornerstone of HIV treatment.

Despite these advances, the high genetic variability of HIV remains a major challenge. This variability is driven by nucleotide substitutions, insertions, deletions, and recombination events, which contribute to genomic diversification [10] and the development of drug resistance [11]. Additionally, the low fidelity of reverse transcriptase [12], along with conformational changes, template switching, and replication dynamics [13], further accelerates the emergence of drug resistance [14,15]. The three-dimensional structure of viral RNA also plays a role in shaping genetic variability and resistance patterns [16].

In this study, we analyzed the genetic diversity of 24 HIV-1 *pol* gene sequences obtained from patients with confirmed HIV-1 infection, along with their associated antiretroviral resistance profiles. Furthermore, structural modeling of HIV-1 protease was performed to explore potential resistance mechanisms using in silico approaches. Among the 24 successfully sequenced samples, 20 were classified as subtype B and four as subtype D, a subtype more commonly reported in African regions. Resistance pattern analysis revealed that only four samples exhibited resistance-associated profiles and were therefore selected for structural modeling. Of these four modeled sequences, three belonged to subtype B and one belonged to subtype D. Remarkably, one sample displayed resistance across all evaluated protease inhibitors (8/8), including five high-level resistance patterns, two intermediate resistance profiles, and one potential low-level resistance profile within the same isolate.

## 2. Materials and Methods

### 2.1. Study Population and Sample Collection

A total of 50 HIV-positive patients, initially diagnosed by enzyme-linked immunosorbent assay (ELISA) and confirmed by Western blot, were included in this study. All patients were recruited from a healthcare institution (IPS de la Costa) in Barranquilla, Colombia. Written informed consent was obtained from all participants, and the study protocol was approved by the Ethics Committee of Universidad Libre, Barranquilla.

Blood samples were collected in EDTA tubes (18 mg K2 EDTA/10 mL; Vacutainer, Ref. 366643) and stored at 4 °C until RNA extraction.

### 2.2. RNA Extraction and RT-PCR Amplification

Blood samples were centrifuged to separate plasma from cellular components. Viral RNA was extracted from plasma using the QIAwave RNA Plus Mini Kit (QIAGEN, Hilden, Germany), following the manufacturer’s instructions.

The HIV-1 *pol* gene was amplified using a one-step RT-PCR approach with the QIAGEN OneStep RT-PCR Kit (QIAGEN, Hilden, Germany) in a final reaction volume of 20 µL. The primers used were forward (5′-TACAGGAGCAGATGATACAG-3′) and reverse (5′-CCTGGCTTTAATTTTACTGG-3′) [17], generating a 266 bp amplicon of the *pol* gene.

Thermal cycling conditions included reverse transcription at 50 °C for 30 min, followed by initial polymerase activation at 95 °C for 15 min, 45 cycles of denaturation at 94 °C for 15 s, annealing at 60 °C for 30 s, and extension at 72 °C for 30 s. Amplification was performed using a CFX96 Touch Real-Time PCR Detection System (Bio-Rad, Hercules, CA, USA).

### 2.3. Sequencing and Phylogenetic Analysis

Sequencing of the protease (PR) gene was performed using the Sanger method with the BigDye Terminator kit (Applied Biosystems, Foster City, CA, USA) on an ABI PRISM 3500 Genetic Analyzer (Applied Biosystems, Foster City, CA, USA).

Sequences were validated using the HIV BLAST database (Los Alamos HIV Sequence Datebase, Los Alamos National Laboratory, Los Alamos, NM, USA, https://www.hiv.lanl.gov, accessed on 20 May 2025), and only sequences with ≥98% similarity to HIV-1 were retained. From the total samples analyzed, 24 sequences were confirmed as HIV-1 and selected for further analysis.

Sequence alignment was performed using ClustalW implemented in MEGA v11 [18], using reference sequences from the Los Alamos HIV database. HIV-1 subtypes were determined using REGA [19], COMET [20], jpHMM-HIV [21], and STAR [22], and subtype assignment was based on concordance in at least three of the four tools.

### 2.4. Antiretroviral Resistance Analysis

Protease gene sequences were analyzed using the Stanford HIV Drug Resistance Database algorithm (version 8.3.4.7) [23]. Identified mutations were confirmed based on the 2019 update of the International Antiviral Society–USA (IAS-USA) mutation list [24]. The prevalence of resistance-associated mutations was assessed according to the Surveillance Drug Resistance Mutations (SDRM) criteria, based on the WHO 2009 mutation list [24]. According to these criteria, the presence of one or more major resistance mutations in treatment-naïve patients was classified as transmitted drug resistance [25].

### 2.5. Structural Modeling

Due to the limited availability of high-resolution crystal structures of HIV-1 protease in the absence of ligands, protein structures were predicted from amino acid sequences derived from samples presenting multiple resistance-associated mutations. Structural modeling was performed using a simplified version of AlphaFold2 version 2.3.2 via ColabFold version 1.6.1 (https://github.com/sokrypton/ColabFold, accessed on 20 May 2025) [26,27]. The highest-confidence models were selected and further prepared using UCSF Chimera version 1.19 [28].

### 2.6. Molecular Docking Analysis

The three-dimensional structure of Nelfinavir (NFV) was obtained from DrugBank (Accession Number: DB00220) and optimized using Avogadro 2.0.0 [29]. Protein–ligand binding sites were predicted using the CB-Dock2 server, which performs blind docking by detecting potential cavities based on solvent-accessible surface clustering and subsequently docking ligands using AutoDock Vina 1.2.7 guided by homologous template patterns The crystallographic structure PDB ID: 2QHC, representing HIV-1 protease in complex with Nelfinavir under the I47A mutation, was retrieved from the Protein Data Bank. Interaction analysis between Nelfinavir and protease was conducted using Discovery Studio v21.1.0.20298 [30]. Both predicted and crystallographic models were structurally aligned using PyMOL [31].

## 3. Results

### 3.1. Phylogenetic Classification of HIV-1 Protease Sequences

To investigate potential mutations associated with antiviral resistance, we focused on codons 35–99 of the HIV-1 protease (*PR*) gene. Amplicons were sequenced by Sanger sequencing.

Sequence data were aligned using ClustalW to confirm viral identity. Of the 50 sequenced samples, 24 were confirmed as HIV-1, while 26 sequences were excluded from further analysis due to lack of concordance.

The 24 confirmed HIV-1 sequences were subsequently analyzed using reference databases to determine circulating subtypes. Among these, 20 sequences were classified as subtype B and 4 as subtype D.

**Figure 1:**
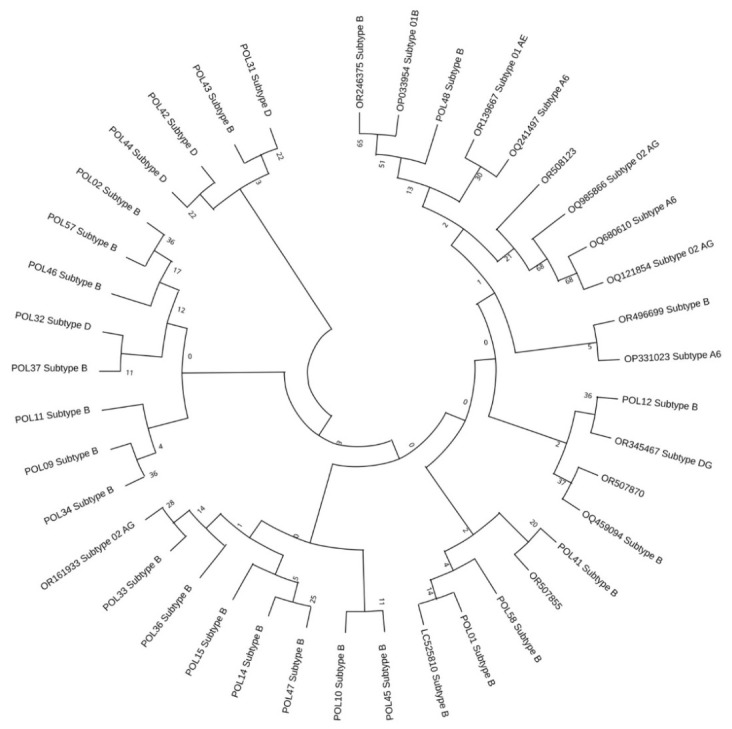
Cladogram based on HIV-1 *pol* gene sequences. The phylogenetic tree was constructed using the Neighbor-Joining method with 1000 bootstrap replicates. Antiretroviral drug resistance patterns were determined.

Genetic distances were estimated by constructing a phylogenetic tree using MEGA (Figure 1). The analysis included reference PR gene sequences retrieved from the Los Alamos National Laboratory database.

The sequence analysis indicated that, despite the relatively small sample size, it was possible to identify circulating molecular patterns associated with resistance to commonly used antiretroviral therapies. The different resistance patterns observed in the analyzed samples are summarized in Table 1.

Based on the resistance profiles summarized in Table 1, four sequences showing some degree of resistance to Nelfinavir were selected for structural modeling. To provide a clearer description of these samples, Table 2 summarizes the protease inhibitor resistance-associated mutations identified in each sequence. Among them, POL09, POL46, and POL48 belonged to subtype B, whereas POL32 belonged to subtype D.

### 3.2. Modeling of Wild-Type Sequences

The role of Nelfinavir in targeting HIV-1 protease (PR) is well established (33). However, based on the findings from the analysis of antiretroviral drug resistance patterns, molecular docking was performed between PR sequences from the four samples exhibiting some degree of resistance and Nelfinavir.

This antiretroviral drug was selected because it showed the highest diversity of resistance patterns among the analyzed treatments.

**Figure 2:**
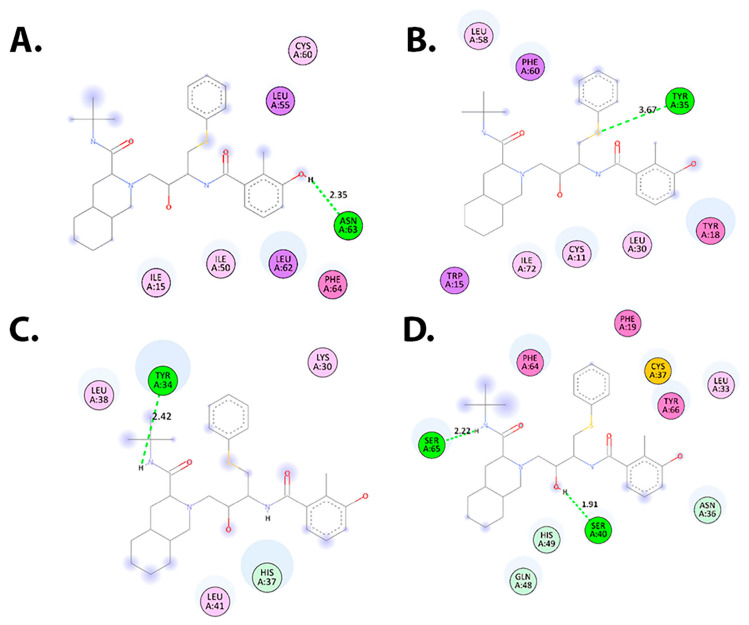
Structural details derived from molecular docking analysis of the interaction between HIV-1 protease (PR) from the four samples exhibiting resistance and Nelfinavir. (**A**). Interaction region of POL09 with Nelfinavir, with a docking score of ΔG: −7.0 kcal/mol. (**B**). Interaction region of POL32 with Nelfinavir, with a docking score of ΔG: −8.6 kcal/mol. (**C**). Interaction region of POL46 with Nelfinavir, with a docking score of ΔG: −5.4 kcal/mol. (**D**). Interaction region of POL48 with Nelfinavir, with a docking score of ΔG: −8.1 kcal/mol. Two-dimensional (2D) representations show the interacting residues, types of interactions, and hydrogen bond distances (Å), depicted as green dashed lines.

Figure 2 illustrates the contact residue analysis of nelfinavir with the PR models derived from samples POL09, POL32, POL46, and POL48, performed using Discovery Studio version 21.1.0.20298. Among these models, POL32 was the only subtype D sequence, whereas POL09, POL46, and POL48 belonged to subtype B. Despite this subtype difference, POL32 showed the same overall non-canonical binding pattern observed in the subtype B resistant sequences, with nelfinavir predicted to bind outside the canonical catalytic pocket.

### 3.3. Structural Analysis of Nelfinavir–Protease Interactions

Based on the structural modeling of HIV-1 protease (PR), four sequences exhibiting molecular resistance patterns to Nelfinavir were selected for further analysis.

In the POL09 sequence, residues Gly49, Ile50, Val82, Asn83, Ile84, Ile85, Gly86, Arg87, Leu90, Cys95, Thr96, Leu97, Asn98, Phe99, and Pro100 were found in direct contact with the drug, forming a predominantly hydrophobic cavity. Nelfinavir established a hydrogen bond interaction with Asn98 involving the H85 atom of the O5 group at a distance of 2.35 Å.

Similarly, in the POL32 sequence, residues Cys46, Asp49, Trp50, Arg51, Tyr53, Ser55, Thr61, Asn64, Leu65, Gln68, Tyr70, Leu93, His94, Phe95, Phe97, Ile107, Lys108, and Ser109 contributed to the formation of a hydrophobic binding cavity. In this case, Nelfinavir interacted with Tyr70 through a hydrogen bond involving the S1 atom at a distance of 3.67 Å, suggesting a relatively weak interaction.

For the POL46 sequence, residues Lys64, Gln67, Tyr68, Asp69, His71, Leu72, Thr74, Leu75, and Ile78 defined the interaction cavity. The drug formed a hydrogen bond with Tyr68 involving the H59 atom of N7 at a distance of 2.42 Å.

Finally, in the POL48 sequence, residues Phe52, Leu66, Asn69, Cys70, Ser73, Thr77, Gln81, His82, Phe95, Lys96, Phe97, Ser98, Tyr99, and Gln100 were involved in the formation of a hydrophobic cavity. Nelfinavir established hydrogen bonds with Ser73 and Ser98 via the H61 atom of O3 and H59 atom of N2, at distances of 1.91 Å and 2.22 Å, respectively.

These findings suggest that Nelfinavir binding within alternative hydrophobic cavities may interfere with its mechanism of action, promoting non-productive interactions that prevent effective blockade of the PR catalytic site. This phenomenon could contribute to the functional neutralization of the inhibitor, allowing the protease to retain its biological activity during viral replication.

To further investigate this mechanism, structural models of HIV-1 protease corresponding to sequences POL09, POL32, POL46, and POL48—previously identified as harboring resistance-associated genotypic patterns—were generated using AlphaFold2. The selected models exhibited high global structural confidence and preserved the canonical architecture of the viral protease.

Subsequently, blind molecular docking was performed between the protease models and Nelfinavir using the CB-Dock2 server. This approach enabled the identification of potential binding cavities without restricting the analysis to the canonical catalytic site. Docking scores ranged from −5.4 to −8.6 kcal/mol, indicating energetically favorable interactions, though not necessarily functionally inhibitory.

Detailed analysis of binding poses revealed that, across all four sequences, Nelfinavir predominantly localized within peripheral or adjacent hydrophobic cavities rather than the catalytic pocket, without establishing direct interactions with the catalytic dyad Asp25/Asp25′, which is essential for effective protease inhibition. Although residues associated with the flap region, such as Gly49, Ile50, and Val82, were involved in the observed interactions, the orientation of the inhibitor differed substantially from the canonical binding mode described for catalytically inhibitory complexes.

**Figure 3:**
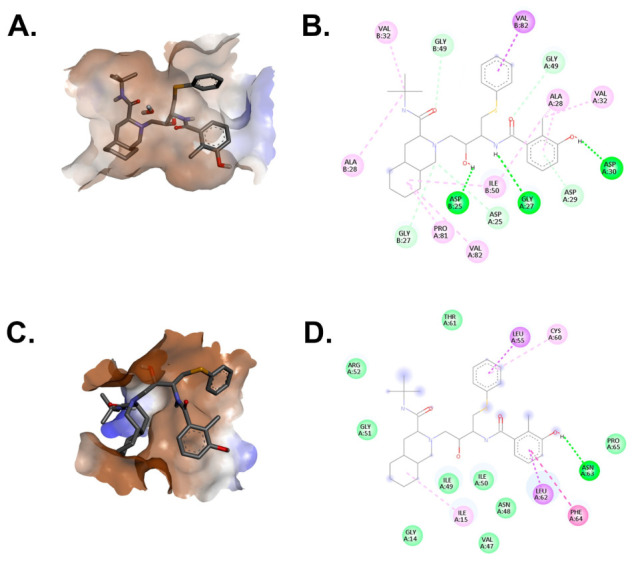
Comparative structural analysis of canonical and non-canonical Nelfinavir binding to HIV-1 protease. (**A**). Three-dimensional representation of the canonical binding mode of Nelfinavir within the hydrophobic catalytic cavity of the wild-type HIV-1 protease, based on the crystallographic complex PDB ID: 1OHR. In this configuration, the inhibitor is positioned within the active-site cleft. (**B**). Two-dimensional interaction map of the canonical PR–Nelfinavir complex, showing the amino acid residues involved in stabilizing the inhibitor within the catalytic binding environment. (**C**). Predicted non-canonical binding pose of Nelfinavir in the resistance-associated POL09 protease variant, showing displacement of the inhibitor toward an alternative hydrophobic cavity outside the canonical catalytic pocket. (**D**). Two-dimensional interaction map of the POL09–Nelfinavir complex, showing a distinct interaction profile involving peripheral non-catalytic residues.

Comparison with the reference crystal structure of the wild-type HIV-1 protease–Nelfinavir complex (PDB ID: 1OHR) showed that, in the canonical binding mode, the inhibitor is deeply positioned within the catalytic cleft and stabilized within the active-site environment, including interactions involving the Asp25/Asp25′ catalytic dyad. This canonical interaction pattern is represented in Figure 3A, while the corresponding two-dimensional interaction map is shown in Figure 3B. In contrast, the POL09 protease variant displayed a displacement of Nelfinavir toward an alternative hydrophobic cavity outside the canonical catalytic pocket, as shown in Figure 3C. The 2D interaction diagram confirmed a distinct network of contacts with peripheral non-catalytic residues (Figure 3D). This pattern suggests that resistance-associated protease variants may favor the stabilization of Nelfinavir in non-catalytic regions, leading to structurally stable but potentially non-productive interactions.

To complement the comparative structural analysis shown in Figure 3, the predicted nelfinavir–protease interaction models for the remaining resistant sequences are provided in Supplementary Figure S1. This supplementary figure shows the binding orientations of nelfinavir in the POL32, POL46, and POL48 protease models, further supporting the observation that nelfinavir was preferentially positioned within peripheral or adjacent hydrophobic cavities rather than within the canonical catalytic pocket.

Overall, these results indicate that, in protease sequences associated with resistance, Nelfinavir may be sequestered within alternative hydrophobic pockets, preventing its proper orientation within the active site and contributing to the loss of inhibitory efficacy observed at the phenotypic level.

## 4. Discussion

Computer-assisted molecular docking, which allows the characterization of binding interactions and conformational geometries between ligands and well-defined molecular targets, has played a key role in the development and optimization of therapeutic strategies against human immunodeficiency virus (HIV) infection. Key targets include viral attachment and fusion processes with host cells [32]. These include interactions with the CD4 receptor and co-receptors [33,34], as well as key enzymes involved in viral genome replication, such as reverse transcriptase, integrase, and protease [35,36,37,38], in addition to processes related to viral assembly and maturation [39].

Resistance patterns to antiretroviral drugs arise primarily from selective pressure exerted during prolonged infection, as well as from abrupt interruptions of therapy [40,41]. Although various resistance-associated patterns linked to changes in viral protein sequences have been identified, the underlying mechanisms by which HIV develops resistance to current drugs are not yet fully understood [42,43].

Molecular interactions between HIV-1 protease and Nelfinavir have been extensively characterized through structural studies, molecular dynamics simulations, and analyses of resistance-associated mutations. Nelfinavir binds to the active site of HIV-1 protease, forming key interactions with residues such as Asp25, Asp30, and other residues within the flap region and catalytic cavity, thereby inhibiting viral maturation [42,43].

The D30N mutation, specifically selected under Nelfinavir pressure, disrupts the direct interaction between the inhibitor and the protease, reducing binding affinity and conferring resistance. Crystallographic studies have shown that the loss of the hydrogen bond between the amide group of Asn30 and Nelfinavir decreases binding energy, explaining both the clinically observed resistance and the reduced catalytic activity of the mutated enzyme [42,43]. Furthermore, the D30N mutation does not confer significant cross-resistance to other protease inhibitors, highlighting the specificity of its interaction with Nelfinavir [42].

Other mutations, such as L90M and combinations like D30N/N88D, also alter the conformational dynamics of the protease and the thermodynamics of Nelfinavir binding, primarily affecting entropic contributions and the structural flexibility of the enzyme–inhibitor complex [44]. Thermodynamic analyses indicate that Nelfinavir binding to mutated proteases is less favorable from an entropic standpoint compared to the wild-type enzyme [44].

Recent molecular dynamics and docking studies further support that mutations such as D30N, L76V, G48T/L89M, and V77I modify the conformation of the active site and the flexibility of the flaps, ultimately impacting Nelfinavir affinity and contributing to resistance. In particular, the D30N mutation introduces electrostatic repulsion with functional groups of Nelfinavir, displacing the drug toward the flap region and promoting flap opening, thereby reducing inhibitory efficacy [45,46,47]. In contrast, mutations such as L76V do not directly disrupt Nelfinavir binding, allowing the drug to retain favorable compatibility with the mutated enzyme [45].

In this study, the results obtained from structural modeling of HIV-1 protease reveal interaction patterns between Nelfinavir and specific enzyme residues that differ substantially from the canonical binding mode described for this inhibitor [48]. Under susceptible conditions, Nelfinavir binds to the catalytic site of the protease, directly interacting with the Asp25/Asp25′ dyad [49,50] and establishing additional contacts with flap residues such as Ile47, Gly48, and Gly49 [50]. This interaction enables a stable orientation of the drug along the catalytic axis, thereby preventing the processing of viral polyproteins.

In contrast, modeling of the POL09, POL32, POL46, and POL48 sequences shows that Nelfinavir predominantly associates with peripheral or adjacent residues rather than the catalytic site, without establishing direct interactions with Asp25/Asp25′. Although some flap residues, such as Gly49, are involved in the observed interactions, the orientation of the inhibitor differs from that required for effective catalytic inhibition. This pattern suggests that, in the analyzed sequences, drug binding occurs outside the functional axis of the enzyme. Although four of the 24 confirmed HIV-1 sequences were classified as subtype D, only one of the four resistant sequences selected for structural modeling belonged to this subtype. Despite being the only subtype D sequence among the resistant models, POL32 did not show a clearly distinct binding pattern compared with the subtype B resistant sequences, since nelfinavir was also predicted to bind outside the canonical catalytic pocket. Therefore, the non-canonical binding behavior observed in our models appears to be more closely related to the resistance-associated mutational background than to subtype assignment alone. Nevertheless, natural amino acid differences among HIV-1 subtypes may influence protease structure, accessory resistance pathways, or inhibitor interactions. For this reason, additional studies including a larger number of subtype D sequences are required to determine whether subtype-specific structural features contribute to altered nelfinavir binding.

Comparison of intermolecular interactions further supports this functional distinction. In the canonical catalytic binding mode [48], Nelfinavir forms short, well-oriented hydrogen bonds [50] with catalytic residues, resulting in high affinity and efficient inhibition. In contrast, in our models, hydrogen bonds are formed with non-catalytic residues and, in some cases, at distances exceeding those considered optimal for functional interactions, as observed in the POL32 sequence. These suboptimal interactions may reflect stable yet non-inhibitory binding.

Additionally, structural modeling revealed the presence of alternative hydrophobic cavities that facilitate the stabilization of Nelfinavir in regions distinct from the active site. This finding contrasts with the canonical binding mode, in which the inhibitor occupies the catalytic pocket and directly blocks proteolytic activity. The presence of these alternative pockets suggests a resistance mechanism based on drug sequestration into conformations that do not interfere with the catalytic function of the protease.

Overall, the contrast between the canonical catalytic binding of Nelfinavir and the interaction patterns observed in this study suggests that the analyzed sequences exhibit a structural resistance mechanism characterized by the displacement of the inhibitor toward non-catalytic hydrophobic cavities. This mechanism likely limits the inhibitory efficacy of Nelfinavir by preventing its proper orientation and interaction with the catalytic dyad, allowing the protease to retain its function during the viral replication cycle. Although these findings are based on modeling approaches, they provide relevant evidence supporting alternative resistance mechanisms that complement those classically described for protease inhibitors.

Although nelfinavir is no longer widely used in current HIV treatment regimens, its interaction with HIV-1 protease remains a useful structural reference for understanding mechanisms of protease inhibitor resistance. In the present study, nelfinavir was not evaluated as a candidate for current therapeutic repositioning, but rather as a well-characterized inhibitor that allows comparison between canonical active-site binding and altered non-canonical binding patterns in resistant protease variants. Therefore, the relevance of these findings lies in their mechanistic contribution to the understanding of how resistance-associated mutations may redirect inhibitor binding away from the catalytic site. Future studies should extend this approach to newer-generation protease inhibitors with greater current clinical use.

## Figures and Tables

**Table 1:** Summary of antiretroviral drug resistance patterns identified in the analyzed samples. Resistance profiles correspond to a region of the HIV-1 pol gene. Abbreviations: Atazanavir (ATV), Darunavir (DRV), Fosamprenavir (FPV), Indinavir (IDV), Lopinavir (LPV), Nelfinavir (NFV), Saquinavir (SQV), Tipranavir (TPV); S, susceptible; LLR, low-level resistance; PLLR, potential low-level resistance; IR, intermediate resistance; HLR, high-level resistance.

ID	Subtipo	ATV	DRV	FPV	IDV	LPV	NFV	SQV	TPV
POL01	B	S	S	S	S	S	S	S	S
POL02	B	S	S	S	S	S	S	S	S
POL09	B	PLLR	S	PLLR	PLLR	S	LLR	S	S
POL10	B	S	S	S	S	S	S	S	S
POL11	B	S	S	S	S	S	S	S	S
POL12	B	S	S	S	S	S	S	S	S
POL14	B	S	S	S	S	S	S	S	S
POL15	B	S	S	S	S	S	S	S	S
POL31	D	S	S	S	S	S	S	S	S
POL32	D	PLLR	PLLR	IR	LLR	LLR	LLR	S	IR
POL33	B	S	S	S	S	S	S	S	S
POL34	B	S	S	S	S	S	S	S	S
POL36	B	S	S	S	S	S	S	S	S
POL37	B	S	S	S	S	S	S	S	S
POL41	B	S	S	S	S	S	S	S	S
POL42	D	S	S	S	S	S	S	S	S
POL43	B	S	S	S	S	S	S	S	S
POL44	D	S	S	S	S	S	S	S	S
POL45	B	S	S	S	S	S	S	S	S
POL46	B	S	S	S	S	S	PLLR	S	LLR
POL47	B	S	S	S	S	S	S	S	S
POL48	B	IR	PLLR	HLR	IR	HLR	HLR	HLR	HLR
POL57	B	S	S	S	S	S	S	S	S
POL58	B	S	S	S	S	S	S	S	S

**Table 2:** Protease inhibitor resistance-associated mutations. Resistance profiles correspond to a region of the HIV-1 pol gene. Mutations were interpreted using the Stanford HIV Drug Resistance Database and cross-checked against the IAS-USA protease inhibitor resistance mutation list.

ID	Subtype	PI Resistance-Associated Mutations Identified by Stanford HIVdb	IAS-USA-Defined PI Resistance Mutations Observed	Additional Rare or Unusual PI-Associated Variants	NFV Resistance Profile
POL09	B	M46V	None	M46V	LLR
POL32	D	I47V, M46N	None	M46N	LLR
POL46	B	Q58E	None	None	PLLR
POL48	B	M46L, I47A, G48A, V82L, I50C	M46L, I47A, V82L	G48A, I50C	HLR

Abbreviations: PI, protease inhibitor; IAS-USA, International Antiviral Society-USA; NFV, Nelfinavir; LLR, low-level resistance; PLLR, potential low-level resistance; HLR, high-level resistance. Note: Only PI resistance-associated mutations are shown. Additional non-resistance mutations identified in the protease sequences are not included in this table.

## Data Availability

The raw data supporting the conclusions of this article will be made available by the authors on request.

## Supplementary Materials

The following supporting information can be downloaded at: https://www.mdpi.com/article/10.3390/v18070701/s1. Supplementary Figure S1. Predicted nelfinavir–protease interaction models for POL32, POL46, and POL48.

## References

[1] Greene W.C. (2007). A History of AIDS: Looking Back to See Ahead. Eur. J. Immunol..

[2] Fischl M.A., Richman D.D., Grieco M.H., Gottlieb M.S., Volberding P.A., Laskin O.L., Leedom J.M., Groopman J.E., Mildvan D., Schooley R.T. (1987). The Efficacy of Azidothymidine (AZT) in the Treatment of Patients with AIDS and AIDS-Related Complex. N. Engl. J. Med..

[3] Rooke R., Tremblay M., Soudeyns H., DeStephano L., Yao X.J., Fanning M., Montaner J.S., O’Shaughnessy M., Gelmon K., Tsoukas C. (1989). Isolation of Drug-Resistant Variants of HIV-1 from Patients on Long-Term Zidovudine Therapy. Canadian Zidovudine Multi-Centre Study Group. AIDS.

[4] Lederman M.M., Connick E., Landay A., Kuritzkes D.R., Spritzler J., St Clair M., Kotzin B.L., Fox L., Chiozzi M.H., Leonard J.M. (1998). Immunologic Responses Associated with 12 Weeks of Combination Antiretroviral Therapy Consisting of Zidovudine, Lamivudine, and Ritonavir: Results of AIDS Clinical Trials Group Protocol 315. J. Infect. Dis..

[5] Ledergerber B., Egger M., Opravil M., Telenti A., Hirschel B., Battegay M., Vernazza P., Sudre P., Flepp M., Furrer H. (1999). Clinical Progression and Virological Failure on Highly Active Antiretroviral Therapy in HIV-1 Patients: A Prospective Cohort Study. Swiss HIV Cohort Study. Lancet.

[6] Murray J.S., Elashoff M.R., Iacono-Connors L.C., Cvetkovich T.A., Struble K.A. (1999). The Use of Plasma HIV RNA as a Study Endpoint in Efficacy Trials of Antiretroviral Drugs. AIDS.

[7] Cesarman E., Damania B., Krown S.E., Martin J., Bower M., Whitby D. (2019). Kaposi Sarcoma. Nat. Rev. Dis. Prim..

[8] Egger M., May M., Chêne G., Phillips A.N., Ledergerber B., Dabis F., Costagliola D., D’Arminio Monforte A., de Wolf F., Reiss P. (2002). Prognosis of HIV-1-Infected Patients Starting Highly Active Antiretroviral Therapy: A Collaborative Analysis of Prospective Studies. Lancet.

[9] Ray M., Logan R., Sterne J.A.C., Hernández-Díaz S., Robins J.M., Sabin C., Bansi L., van Sighem A., de Wolf F., HIV-CAUSAL Collaboration (2010). The Effect of Combined Antiretroviral Therapy on the Overall Mortality of HIV-Infected Individuals. AIDS.

[10] Hu W.-S., Hughes S.H. (2012). HIV-1 Reverse Transcription. Cold Spring Harb. Perspect. Med..

[11] Charpentier C., Nora T., Tenaillon O., Clavel F., Hance A.J. (2006). Extensive Recombination among Human Immunodeficiency Virus Type 1 Quasispecies Makes an Important Contribution to Viral Diversity in Individual Patients. J. Virol..

[12] Beard W.A., Wilson S.H. (1994). Site-Directed Mutagenesis of HIV Reverse Transcriptase to Probe Enzyme Processivity and Drug Binding. Curr. Opin. Biotechnol..

[13] Dash C. (2004). Using Pyrrolo-Deoxycytosine to Probe RNA/DNA Hybrids Containing the Human Immunodeficiency Virus Type-1 3′ Polypurine Tract. Nucleic Acids Res..

[14] Abram M.E., Ferris A.L., Shao W., Alvord W.G., Hughes S.H. (2010). Nature, Position, and Frequency of Mutations Made in a Single Cycle of HIV-1 Replication. J. Virol..

[15] Goody R.S., Müller B., Restle T. (1991). Factors Contributing to the Inhibition of HIV Reverse Transcriptase by Chain-Terminating Nucleotides in Vitro and in Vivo. FEBS Lett..

[16] Brady S., Singh G., Bolinger C., Song Z., Boeras I., Weng K., Trent B., Brown W.C., Singh K., Boris-Lawrie K. (2019). Virion-Associated, Host-Derived DHX9/RNA Helicase A Enhances the Processivity of HIV-1 Reverse Transcriptase on Genomic RNA. J. Biol. Chem..

[17] Goffin V. (2005). Transcription Factor Binding Sites in the Pol Gene Intragenic Regulatory Region of HIV-1 Are Important for Virus Infectivity. Nucleic Acids Res..

[18] Tamura K., Stecher G., Kumar S. (2021). MEGA11: Molecular Evolutionary Genetics Analysis Version 11. Mol. Biol. Evol..

[19] Pineda-Peña A.-C., Faria N.R., Imbrechts S., Libin P., Abecasis A.B., Deforche K., Gómez-López A., Camacho R.J., de Oliveira T., Vandamme A.-M. (2013). Automated Subtyping of HIV-1 Genetic Sequences for Clinical and Surveillance Purposes: Performance Evaluation of the New REGA Version 3 and Seven Other Tools. Infect. Genet. Evol..

[20] Struck D., Perez-Bercoff D., Devaux C., Schmit J.C., Danielle P.B. COMET: A Novel Approach to HIV-1 Subtype Prediction. Proceedings of the 8th European HIV Drug Resistance Workshop.

[21] Schultz A.-K., Zhang M., Bulla I., Leitner T., Korber B., Morgenstern B., Stanke M. (2009). jpHMM: Improving the Reliability of Recombination Prediction in HIV-1. Nucleic Acids Res..

[22] Myers: A Statistical Model for HIV-1 Sequence Classificat…—Google Académico. https://academic.oup.com/bioinformatics/article/21/17/3535/212806.

[23] Liu T.F., Shafer R.W. (2006). Web Resources for HIV Type 1 Genotypic-Resistance Test Interpretation. Clin. Infect. Dis..

[24] Wensing A.M., Calvez V., Ceccherini-Silberstein F., Charpentier C., Günthard H.F., Paredes R., Shafer R.W., Richman D.D. (2019). 2019 Update of the Drug Resistance Mutations in HIV-1. Top. Antivir. Med..

[25] Bennett D.E., Camacho R.J., Otelea D., Kuritzkes D.R., Fleury H., Kiuchi M., Heneine W., Kantor R., Jordan M.R., Schapiro J.M. (2009). Drug Resistance Mutations for Surveillance of Transmitted HIV-1 Drug-Resistance: 2009 Update. PLoS ONE.

[26] Jumper J., Evans R., Pritzel A., Green T., Figurnov M., Ronneberger O., Tunyasuvunakool K., Bates R., Žídek A., Potapenko A. (2021). Highly Accurate Protein Structure Prediction with AlphaFold. Nature.

[27] Varadi M., Anyango S., Deshpande M., Nair S., Natassia C., Yordanova G., Yuan D., Stroe O., Wood G., Laydon A. (2022). AlphaFold Protein Structure Database: Massively Expanding the Structural Coverage of Protein-Sequence Space with High-Accuracy Models. Nucleic Acids Res..

[28] Pettersen E.F., Goddard T.D., Huang C.C., Couch G.S., Greenblatt D.M., Meng E.C., Ferrin T.E. (2004). UCSF Chimera—A Visualization System for Exploratory Research and Analysis. J. Comput. Chem..

[29] Hanwell M.D., Curtis D.E., Lonie D.C., Vandermeersch T., Zurek E., Hutchison G.R. (2012). Avogadro: An Advanced Semantic Chemical Editor, Visualization, and Analysis Platform. J. Cheminform..

[30] Dassault Systèmes BIOVIA (2021). Discovery Studio.

[31] (2015). The PyMOL Molecular Graphics System.

[32] Debnath A.K. (2013). Rational Design of HIV-1 Entry Inhibitors. Methods Mol. Biol..

[33] Yu F., Jiang S. (2022). Small-Molecule HIV Entry Inhibitors Targeting Gp120 and Gp41. Adv. Exp. Med. Biol..

[34] Kellenberger E., Springael J.-Y., Parmentier M., Hachet-Haas M., Galzi J.-L., Rognan D. (2007). Identification of Nonpeptide CCR5 Receptor Agonists by Structure-Based Virtual Screening. J. Med. Chem..

[35] Jadhav A.K., Karuppayil S.M. (2021). Andrographis Paniculata (Burm. F) Wall Ex Nees: Antiviral Properties. Phytother. Res..

[36] Dharmalingam T., Udhaya V., Umaarasu T., Elangovan V., Rajesh S.V., Shanmugam G. (2015). Prediction of Drug-Resistance Using Genotypic and Docking Analysis Among Anti-Retroviral Therapy Naïve and First-Line Treatment Failures in Salem, Tamil Nadu, India. Curr. HIV Res..

[37] Miceli L., Teixeira V., Castro H., Rodrigues C., Mello J., Albuquerque M., Cabral L., De Brito M., De Souza A. (2013). Molecular Docking Studies of Marine Diterpenes as Inhibitors of Wild-Type and Mutants HIV-1 Reverse Transcriptase. Mar. Drugs.

[38] Tintori C., Corona A., Esposito F., Brai A., Grandi N., Ceresola E.R., Clementi M., Canducci F., Tramontano E., Botta M. (2016). Inhibition of HIV-1 Reverse Transcriptase Dimerization by Small Molecules. ChemBioChem.

[39] Olotu F.A., Agoni C., Soremekun O., Soliman M.E.S. (2020). The Recent Application of 3D-QSAR and Docking Studies to Novel HIV-Protease Inhibitor Drug Discovery. Expert. Opin. Drug Discov..

[40] Zuo Z., Huang W., Liu L., Li M., Chen H., Feng Y., Liao L., Shao Y., Ruan Y., Wu J. (2025). HIV Drug Resistance and Its Associated Factors among Patients during Interruption of Antiretroviral Therapy in China. Front. Microbiol..

[41] Geremia N., Basso M., De Vito A., Scaggiante R., Giobbia M., Battagin G., Dal Bello F., Giordani M.T., Nardi S., Malena M. (2024). Patterns of Transmitted Drug Resistance Mutations and HIV-1 Subtype Dynamics in ART-Naïve Individuals in Veneto, Italy, from 2017 to 2024. Viruses.

[42] Bihani S.C., Das A., Prashar V., Ferrer J.-L., Hosur M.V. (2009). Resistance Mechanism Revealed by Crystal Structures of Unliganded Nelfinavir-Resistant HIV-1 Protease Non-Active Site Mutants N88D and N88S. Biochem. Biophys. Res. Commun..

[43] Antunes D.A., Rigo M.M., Sinigaglia M., De Medeiros R.M., Junqueira D.M., Almeida S.E.M., Vieira G.F. (2014). New Insights into the In Silico Prediction of HIV Protease Resistance to Nelfinavir. PLoS ONE.

[44] Kožíšek M., Bray J., Řezáčová P., Šašková K., Brynda J., Pokorná J., Mammano F., Rulíšek L., Konvalinka J. (2007). Molecular Analysis of the HIV-1 Resistance Development: Enzymatic Activities, Crystal Structures, and Thermodynamics of Nelfinavir-Resistant HIV Protease Mutants. J. Mol. Biol..

[45] Pandarinathan S., Jayanthi S. (2026). Insights into the Interaction Mechanism of First-Generation HIV-1 Protease Inhibitors with Wild-Type and Mutant (D30N and L76V) Enzymes through in-Silico Based Approach. J. Biomol. Struct. Dyn..

[46] Wang R.-G., Zhang H.-X., Zheng Q.-C. (2020). Revealing the Binding and Drug Resistance Mechanism of Amprenavir, Indinavir, Ritonavir, and Nelfinavir Complexed with HIV-1 Protease Due to Double Mutations G48T/L89M by Molecular Dynamics Simulations and Free Energy Analyses. Phys. Chem. Chem. Phys..

[47] Gupta A., Jamal S., Goyal S., Jain R., Wahi D., Grover A. (2015). Structural Studies on Molecular Mechanisms of Nelfinavir Resistance Caused by Non-Active Site Mutation V77I in HIV-1 Protease. BMC Bioinform..

[48] Wlodawer A., Miller M., Jaskólski M., Sathyanarayana B.K., Baldwin E., Weber I.T., Selk L.M., Clawson L., Schneider J., Kent S.B.H. (1989). Conserved Folding in Retroviral Proteases: Crystal Structure of Synthetic HIV-1 Protease. Science.

[49] Shen C.-H., Wang Y.-F., Kovalevsky A.Y., Harrison R.W., Weber I.T. (2010). Amprenavir Complexes with HIV-1 Protease and Its Drug-Resistant Mutants Altering Hydrophobic Clusters. FEBS J..

[50] Perez M.A.S., Fernandes P.A., Ramos M.J. (2007). Drug Design: New Inhibitors for HIV-1 Protease Based on Nelfinavir as Lead. J. Mol. Graph. Model..

