# Supplementary material for: Non-Canonical Binding of Nelfinavir in HIV-1 Protease Variants Reveals Structural Mechanisms of Antiretroviral Resistance
Source: Viruses. 2026 Jun 25;18(7):701. doi: 10.3390/v18070701 (PMC13431487; doi:10.3390/v18070701)

Supplemetary material

Supplementary Figure S1. Predicted nelfinavir–protease interaction models for POL32, POL46, and POL48. Three-dimensional and two-dimensional representations of the predicted interaction between nelfinavir and HIV-1 protease models are shown for the three remaining resistant sequences analyzed. **A.** Three-dimensional binding orientation of nelfinavir in the POL32 protease model. **B.** Two-dimensional interaction map of the POL32–nelfinavir complex. **C.** Three-dimensional binding orientation of nelfinavir in the POL46 protease model. **D.** Two-dimensional interaction map of the POL46–nelfinavir complex. **E.** Three-dimensional binding orientation of nelfinavir in the POL48 protease model. **F.** Two-dimensional interaction map of the POL48–nelfinavir complex. These models complement the comparative analysis shown in Figure 3 and illustrate the predicted non-canonical binding orientations of nelfinavir in peripheral or adjacent hydrophobic cavities rather than within the canonical catalytic pocket.

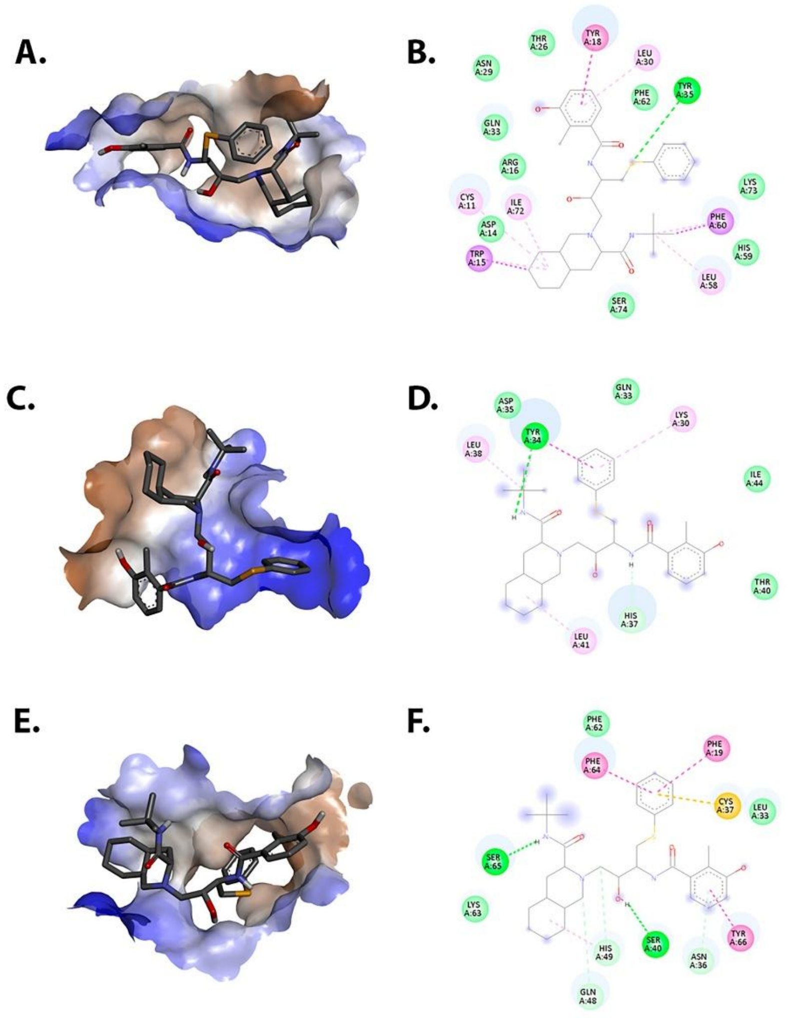

Supplement: Supplementary file 1 [file viruses-18-00701-s001.zip › viruses-4364424-supplementary.pdf]
